# Five differentially expressed proteins identified to serve as potential blood biomarkers for schizophrenia screening based on proteomics

**DOI:** 10.3389/fpsyt.2025.1697383

**Published:** 2026-01-07

**Authors:** Ronghua Li, Xiaoqian Fu, Chuanwei Li, Lian Yuan, Yaozhi Liu, Lin Yang, Xiaojia Fang, Xiaobin Zhang, Guangya Zhang, Xiangdong Du

**Affiliations:** 1 Suzhou Guangji Hospital, The Affiliated Guangji Hospital of Soochow University, Suzhou, China; 2 Medical College of Soochow University, Suzhou, China; 3 School of Psychiatry, North Sichuan Medical College, Nanchong, Sichuan, China; 4 Affiliated Mental Health Center and Hangzhou Seventh People’s Hospital, Zhejiang University School of Medicine, Hangzhou, Zhejiang, China

**Keywords:** schizophrenia, proteomics, biomarkers, mass spectrometry, mitogen-activated protein kinase signaling pathway

## Abstract

**Introduction:**

Schizophrenia is a multifactorial neuropsychiatric disorder characterized by a wide range of debilitating symptoms and relatively poor clinical outcome, bringing a huge burden of disease. However, the underlying pathological mechanism of this disease remains unclear. We aimed to use mass spectrometry to complete proteomics analysis to find biomarkers related to schizophrenia in peripheral blood, which can provide some biomarker for the pathology, diagnosis or treatment of schizophrenia and the focus of future research.

**Methods:**

This study was a cross-sectional case-control study involving 46 patients with schizophrenia and 43 healthy controls. All subjects provided early morning fasting cubital venous blood, completed the collection of general demographic data and clinical scale evaluation. At last, the blood samples of all subjects were subjected to mass spectrometry analysis, combined with bioinformatics analysis, to identify and screen differential proteins.

**Results:**

Using nominal P<0.05 (uncorrected) and fold change > 1.5 or < 0.67, 34 proteins were prioritized, among which 22 proteins were up-regulated and 12 were down-regulated; these candidates require false discovery rate controlled verification. Gene ontology over-representation analysis suggested trends in cellular component organization and cell adhesion molecule binding, with no false discovery rate correction. Kyoto encyclopedia of genes and genomes functional enrichment analysis showed that the differential proteins were mainly involved in mitogen-activated protein kinase signaling pathways.

**Discussion:**

Our research indicates that neural cell adhesion molecule L1, integrin alpha-M, alpha-actinin-1, filamin-A and profilin-1 which are associated with cytoskeleton, synapse and immunity were preliminarily screened as candidate protein markers for schizophrenia. Moreover, mitogen-activated protein kinase signaling pathway may be related to the pathology of schizophrenia, and PI3K-Akt signaling pathway may be related to the efficacy and side effects of antipsychotic drugs.

## 1 Introduction

Schizophrenia is one of the common and serious mental disorders, affecting nearly 1% of the world’s population (1). It was ranked among the top 15 leading causes of disability in the world in 2016 (2), bringing a huge burden of disease (3, 4). Its main clinical manifestations include positive psychiatric symptoms such as hallucinations, delusions, and speech disorders; negative symptoms such as decreased motivation and decreased expression ability; and cognitive function defects involving executive function and memory (5). The disease typically starts in young adults, has a protracted course, seriously affecting the social function and quality of life of patients.

Biomarkers refer to indicators that can be objectively measured and evaluated, which are usually divided into three categories: 1) those that can reflect the occurrence and development process of diseases; 2) those that can evaluate the effect of drugs; and 3) those that can be used as indicators of clinical treatment endpoints (6). The diagnosis of schizophrenia is mainly dependent on clinical doctors evaluating the mental symptoms and diagnostic level affected by individual ability. In addition, there is a lack of corresponding clinical evaluation system for the prognosis of patients; therefore, discovering objective and effective biomarkers is an urgent clinical issue that needs to be solved. The pathogenesis of schizophrenia may involve various aspects, such as neuroimaging, biochemistry, genetics, and neuro-electrophysiology, and the research methods are also diverse. In recent years, proteomics has become one of the most popular research methods.

The word “proteomics” was proposed for the first time in 1995 (7–9). It refers to the large-scale and global analysis of proteins in a system at specific time points and under specific conditions (10). Proteins perform a vast majority of functions in every organism, and the proteome comprises all of the proteins produced or modified by an organism. The aim of proteomics is to obtain a more comprehensive and integrated biological view by studying these proteins at one time (7). Therefore, proteomic analysis can better reflect the dynamic pathophysiological process. With the development of high-throughput technology, mass spectrometry-based proteomics can characterize the human plasma proteome with unprecedented accuracy (11) and can predict not only the onset of disease but also its course and even the outcome (7, 12, 13). The continuous development of mass spectrometry technology has deepened our understanding of the origin of schizophrenia and the various hypotheses regarding this disease, such as the dopaminergic, GABAergic, and neurodevelopmental theories (14). Proteomics provides an overall overview of important information about the physiological state of the cells, tissues, or organisms as it regulates the protein expression at various levels, including transcription, epigenetics, translation, and degradation (15).

Current proteomic studies have found that the pathology of schizophrenia is related to stress response, inflammation, and congenital or acquired immune and energy metabolism processes whose related proteins are expected to become disease biomarkers. However, nothing has been decided yet (16–19). In addition, a study on neuroproteomics discovered that pathways such as spliceosomes and amino acid metabolism, axonal guidance, and synaptogenesis show impairments in patient-derived induced pluripotent stem cells (20). Oraki Kohshour et al. found evidence of cyclic protein analysis being able to identify specific biomarkers for schizophrenia and bipolar disorder (21). Campeau et al. conducted a comparative analysis of the plasma proteome of patients with schizophrenia and normal controls over a 60-year life span. They found increased levels of the biomarkers associated with the physiological comorbidity risk of schizophrenia, such as C-reactive protein and low-density lipoprotein, particularly in younger individuals, and the results based on mass spectrometry proteomic data were significantly correlated with the clinical laboratory measurements (22). Blood is an easily accessible biological sample that can interact with all systems and reflect the physiological and pathological changes of any part of the body to some extent. Hence, more and more scholars have been trying to discover peripheral blood biomarkers of the disease (19, 23, 24). Therefore, this study aimed to determine protein biomarkers related to schizophrenia through mass spectrometry-based proteomic analysis in the peripheral blood of a Chinese Han population in order to provide more references for the pathology, diagnosis, or treatment of this disease.

## 2 Materials and methods

### 2.1 Subjects

In this study, we enrolled a total of 46 patients with schizophrenia (13 women and 33 men) and 43 healthy controls (19 women and 24 men). The median (quartile) age of the patient group was 46.00 years (38.75–52.00 years), while that of the control group was 38.00 years (30.00–51.00 years). The clinical diagnosis of schizophrenia was made by two experienced psychiatrists according to the Diagnostic and Statistical Manual of Mental Disorders, Fifth Edition (DSM-V) criteria. The inclusion criteria for patients were: age 18–65 years, old enough to understand the research content, in a stable condition, can cooperate with the study, and with adequate understanding of the study content and completion of the informed consent process. The inclusion criteria for the healthy control subjects were: age 18–65 years, no history of DSM-5 diagnosis, no family history of mental illness, and with cognitive function able to understand the content of the study while completing the informed consent process. We excluded potential participants with organic brain diseases and other serious body diseases; with abnormal recent blood counts, heart, liver, or kidney function; with a history of other mental illnesses such as intellectual disability and mood disorders; with alcohol or drug dependence; had undergone electroconvulsive therapy; and who were pregnant or lactating women. All of the subjects were recruited from the Han Chinese population in Jiangsu Province. The gender and age between the schizophrenia patients and the healthy controls had no obvious statistical differences (**Table 1**).

**Table 1:** General demographic data and scale assessment in the patient and control groups.

Item	Patient group (*n*=46)	Control group (*n*=43)	*χ*^2^/*Z*/*t*	*p*
Age (years)	46.00 (38.75–52.00)	38.00 (30.00–51.00)	−1.914	0.056
BMI (kg/m^2^)	24.33 ± 3.85	23.98 ± 3.69	0.438	0.662
Sex			2.448	0.118
Men	33 (71.7)	24 (55.8)		
Women	13 (28.3)	19 (44.2)		
Duration of disease (years)	20.52 ± 10.55	–		
Chlorpromazine equivalent (mg)	500.00 (393.75–741.50)	–		
Total PANSS score	49.50 (43.00–56.00)	–		
PANSS positive score	7.00 (7.00–11.00)	–		
PANSS negative score	18.00 (14.75–22.00)	–		
PANSS general pathological score	23.00 (20.00–27.25)	–		

PANSS, Positive and Negative Syndrome Scale.

The study was conducted in accordance with the Declaration of Helsinki and was approved by the Ethics Committee of Suzhou Guangji Hospital. Written informed consent was voluntarily provided by all subjects or their legal guardians.

### 2.2 Study design

Schizophrenia patients and healthy volunteers were enrolled in this case–control study. Firstly, general demographic data including the name, gender, age, and clinical data such as the course of the disease and the dose of antipsychotic drugs were collected using self-made information tables. In the patient group, a total of 5 ml of fasting cubital venous blood was collected in ethylenediaminetetraacetic acid (EDTA) anticoagulant tubes and centrifuged using an Eppendorf centrifuge at 3,000 rpm for 10 min. The separated plasma was placed in aliquots and stored in a −80°C refrigerator. In the control group, blood samples were collected, centrifuged, and frozen.

### 2.3 Determination of proteins

#### 2.3.1 Sample preparation

The samples were mixed with 8 M urea/100 mM Tris–HCl and subjected to treatment with water bath sonication. After centrifugation, the supernatant was used for the reduction reaction (10 mM DTT, 37°C for 1 h), followed by the alkylation reaction (40 mM iodoacetamide, room temperature/dark for 30 min). The protein concentration was measured with the Bradford method. Urea was diluted below 2 M using 100 mM Tris–HCl (pH 8.0). Trypsin was added at a ratio of 1:50 (enzyme/protein, *w*/*w*) for overnight digestion at 37°C. The following day, trifluoroacetic acid (TFA) was used to bring the pH down to 6.0 to end the digestion. After centrifugation (12,000 × *g*, 15 min), the supernatant was subjected to peptide purification using the Sep-Pak C18 desalting column. The peptide eluate was vacuum-dried and stored at −20°C for later use.

#### 2.3.2 Mass spectrometry detection

Mass spectrometry data acquisition was carried out on an Orbitrap Exploris 480 mass spectrometer coupled with an Easy-nLC 1200 system. The peptides were loaded through an autosampler and separated in a C18 analytical column (75 μm × 25 cm, C18, 1.9 μm, 100 Å). Mobile phase A (0.1% formic acid) and mobile phase B [80% acetonitrile (ACN) and 0.1% formic acid] were used to establish the separation gradient. A constant flow rate was set at 300 nl/min. For data-independent acquisition (DIA) mode analysis, each scan cycle consisted of one full-scan mass spectrum [*R*=60K, automatic gain control (AGC)=3*e*6, maximum injection time (maxIT)=30 ms, and scan range=350–1,250 *m*/*z*] followed by 40 variable MS/MS events (*R*=30K, AGC=1,000%, maxIT=50 ms). The high-field asymmetric waveform ion mobility spectrometry (FAIMS) compensation voltage (CV) was set to −45. The higher-energy collision dissociation (HCD) was set to 30.

#### 2.3.3 Protein database search and analysis

The raw mass spectrometry data were processed with the DIA-NN software (v1.7.16) using a library-free method. Firstly, the Human Protein Sequence database from SwissProt (Human, 20210312), which does not include the reviewed isoforms, was used for library prediction using deep learning algrithms. The match between run (MBR) function was employed to create a spectral library from the DIA data and then reanalyzed using this library. The final precursor and protein false discovery rate (FDR) was set as 1% FDR. The output files of DIA-NN containing the quantification information of the protein groups were used for further analysis. The ratio of the repeated quantitative means of each protein in two sets of samples was taken as the fold change (FC). The relative quantitative values of each protein in the two groups of samples were further assessed using a *t*-test to determine the significance of the difference. A corresponding *p*-value was calculated. FC quantifies the relative change between datasets. FC >1.5 or <0.67 and a *p*-value <0.05 were set as the criteria for significant changes between the two groups (25, 26). The differential proteins were displayed with a volcano diagram using R (base package, 3.5.1), and the change trend of the differential proteins was evaluated. Combined with a hypergeometric test, Gene Ontology (GO; http://www.geneontology.org/), the Kyoto Encyclopedia of Genes and Genomes (KEGG; http://www.genome.jp/kegg/) database, and R (clusterProfiler, 3.10.1) were used for functional enrichment analyses of the differential proteins to determine the GO entries and KEGG pathways in which the differential proteins are involved. In addition, the STRING protein interaction database (http://string-db.org/) was used for protein interaction analysis. Firstly, the differential protein sequences were compared with the sequences extracted by BLAST (2.8.1 http://blast.ncbi.nlm.nih.gov/Blast.cgi) to obtain the corresponding protein interaction information. Subsequently, R (igraph, 1.2.4.2) software was used to construct the network graph.

### 2.4 Statistical methods

The Kolmogorov–Smirnov test was used to test for normal distribution when the sample size was greater than 50; otherwise, the Shapiro–Wilk test was used. Measurement data with normal distribution were expressed as the mean ± standard deviation (*x* ± *s*), and an independent-samples *t*-test was used for comparisons between two groups. Quantitative data with a skewed distribution were expressed as median and quartile [*M* (P25–P75)], and comparisons between groups were analyzed using the Mann–Whitney *U* test. The number and percentage of cases were used to describe the count data, and the chi-square test was used for comparisons between groups. Pearson’s correlation analysis was used when two groups of data conform to a normal distribution, while Spearman’s correlation analysis was used when the data are not normally distributed. A two-tailed *p*<0.05 was considered statistically significant.

## 3 Results

### 3.1 General demographic data

A total of 46 patients with schizophrenia and 43 healthy volunteers were enrolled in this study. There were no significant differences in age, gender, and body mass index (BMI) between the two groups (*p* > 0.05). According to the statistics, the median (quartile) age of the patient group was 46.00 years (38.75–52.00 years), while that of the control group was 38.00 years (30.00–51.00 years) (**Table 1**).

### 3.2 Proteomic analysis

#### 3.2.1 Quantitative protein analysis

Based on DIA quantitative proteomics technology, after the database search and the DIA-NN algorithm, the detection results were screened with 1% FDR. The number of total peptides and proteins identified is shown in **Table 2**.

**Table 2:** Protein identification results.

Database	Peptides	Proteins
SwissProt Human (20210312)	7,633	668

The species name was selected from the database used. Shown are the total number of identified peptide segments and the total number of identified proteins.

#### 3.2.2 Differential protein analysis

**Figure 1:**
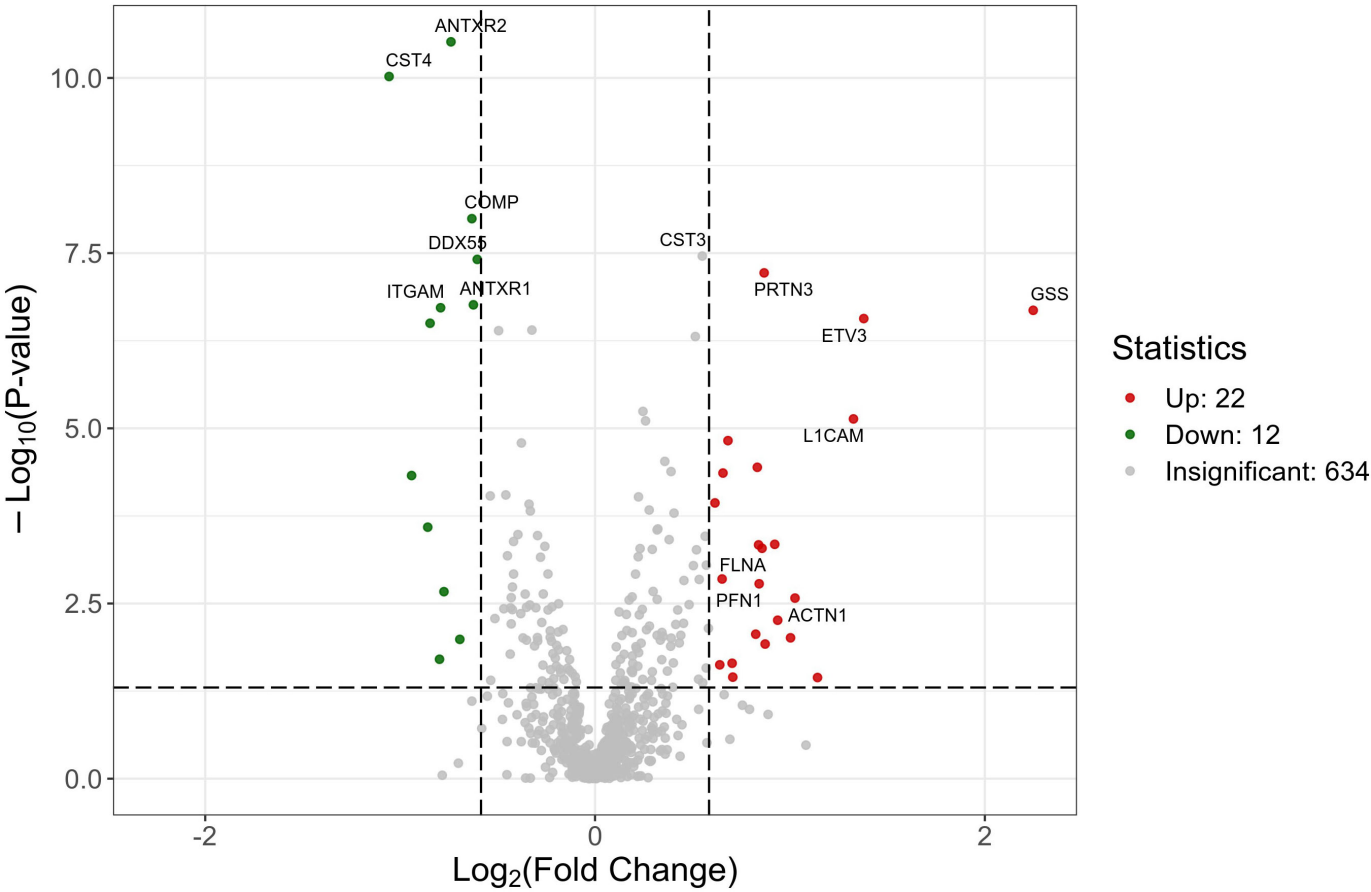
Volcano plot of the differential proteins. The *horizontal axis* shows the fold change (log_2_ value) of the differentially expressed proteins, while the *vertical axis* shows the *p*-value (−log10 value). *Blue* represents the proteins with no significant difference, *red* represents the upregulated proteins, and *green* represents the downregulated proteins.

Through the analysis, a total of 34 differentially expressed proteins were identified and screened. Compared with the healthy control group, there were 22 upregulated proteins and 12 downregulated proteins in the schizophrenia group. These candidate proteins require FDR-controlled verification, as shown in **Table 3**. At the same time, the volcano plot can be used to quickly assess the differences in protein expression levels between two (or more) samples, as well as the statistical significance of these differences. Take the logarithm base 2 for each protein difference ratio, and take the absolute value of the logarithm base 10 of the P-value, and then draw a volcano plot. The volcano map is shown in **Figure 1**.

**Table 3:** Differential proteins in the schizophrenia patient group and the healthy control group.

Protein ID	Protein name	Gene	Log_2_FC	Expression
A0A075B6R2	Immunoglobulin heavy variable 4-4	*IGHV4-4*	0.839153387389821	Up
A0A0C4DH33	Immunoglobulin heavy variable 1-24	*IGHV1-24*	0.656450606	Up
O75717	WD repeat and HMG-box DNA-binding protein 1	*WDHD1*	−0.85830564	Down
P01036	Cystatin-S	*CST4*	−1.0564912	Down
P01880	Immunoglobulin heavy constant delta	*IGHD*	0.706817847	Up
P05164	Myeloperoxidase	*MPO*	0.615322123	Up
P07737	Profilin-1	*PFN1*	0.84213408	Up
P08519	Apolipoprotein(a)	*LPA*	0.639973004	Up
P0DJI8	Serum amyloid A-1 protein	*SAA1*	0.92189329	Up
P11215	Integrin alpha-M	*ITGAM*	−0.79235699	Down
P12814	Alpha-actinin-1	*ACTN1*	1.025950124	Up
P17301	Integrin alpha-2	*ITGA2*	0.832606509	Up
P19013	Keratin, type II cytoskeletal 4	*KRT4*	−0.79786442	Down
P21333	Filamin A	*FLNA*	0.856866207	Up
P24158	Myeloblastin	*PRTN3*	0.867659289	Up
P31946	14-3–3 Protein beta/alpha	*YWHAB*	−0.69362109	Down
P32004	Neural cell adhesion molecule L1	*L1CAM*	1.325846898	Up
P36957	Dihydrolipoyllysine-residue succinyltransferase component of 2-oxoglutarate dehydrogenase complex, mitochondrial	*DLST*	0.682033871	Up
P37802	Transgelin-2	*TAGLN2*	0.872789368	Up
P41162	ETS translocation variant 3	*ETV3*	1.378789038	Up
P48637	Glutathione synthetase	*GSS*	2.248140105	Up
P49747	Cartilage oligomeric matrix protein	*COMP*	−0.63195807	Down
P58335	Anthrax toxin receptor 2	*ANTXR2*	−0.73913126	Down
P61224	Ras-related protein Rap-1b	*RAP1B*	0.824482748	Up
P63104	14-3–3 Protein zeta/delta	*YWHAZ*	0.703366528	Up
P67936	Tropomyosin alpha-4 chain	*TPM4*	0.937118365	Up
Q16270	Insulin-like growth factor-binding protein 7	*IGFBP7*	−0.94119865	Down
Q6ZUL3	Uncharacterized protein C8orf86	*C8orf86*	0.652050466	Up
Q86TH1	ADAMTS-like protein 2	*ADAMTSL2*	−0.77521881	Down
Q86UX7	Fermitin family homolog 3	*FERMT3*	1.003098465	Up
Q8NEP3	Dynein axonemal assembly factor 1	*DNAAF1*	−0.84632435	Down
Q8NHQ9	ATP-dependent RNA helicase DDX55	*DDX55*	−0.60427909	Down
Q9H6X2	Anthrax toxin receptor 1	*ANTXR1*	−0.62415428	Down
Q9H799	Ciliogenesis and planar polarity effector 1	*CPLANE1*	1.141498188	Up

Log_2_FC, log_2_ fold change; Up, upregulated; Down, downregulated.

#### 3.2.3 Differences in protein GO enrichment analysis

**Figure 2:**
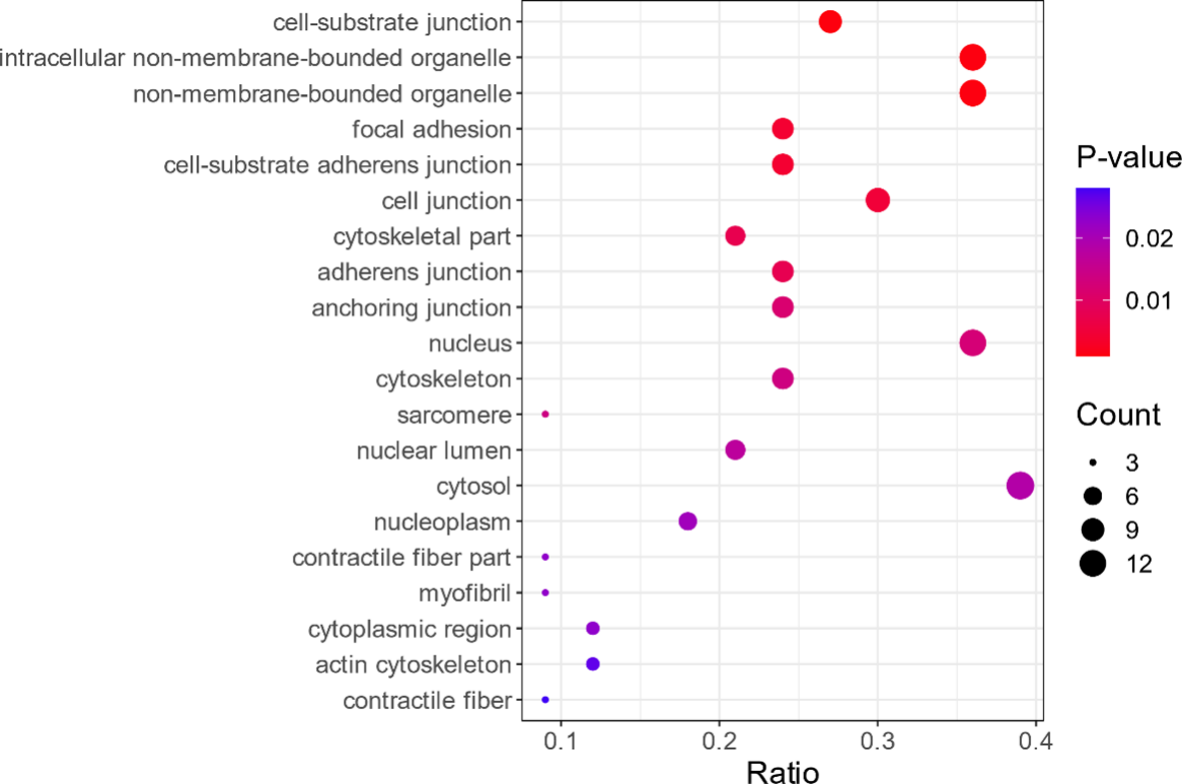
Bubble plot of the Gene Ontology (GO) enrichment analysis in terms of cellular components (only the top 20 are shown). The *abscissa* represents the ratio between the number of differential proteins in the corresponding GO entry and the number of all proteins identified in the GO entry. The higher the value, the higher the enrichment degree of the differential proteins in the GO entry. The *color of the dot*, ranging from *blue* to *red*, indicates the *p*-value of the hypergeometric test. The *redder the color*, the smaller the *p*-value, the greater the reliability of the test, and the more statistically significant is the result. The *size of the dot* indicates the number of differentially expressed proteins in the corresponding GO entry. The *larger the dot*, the greater the number of differentially expressed proteins in the GO entry.

**Figure 3:**
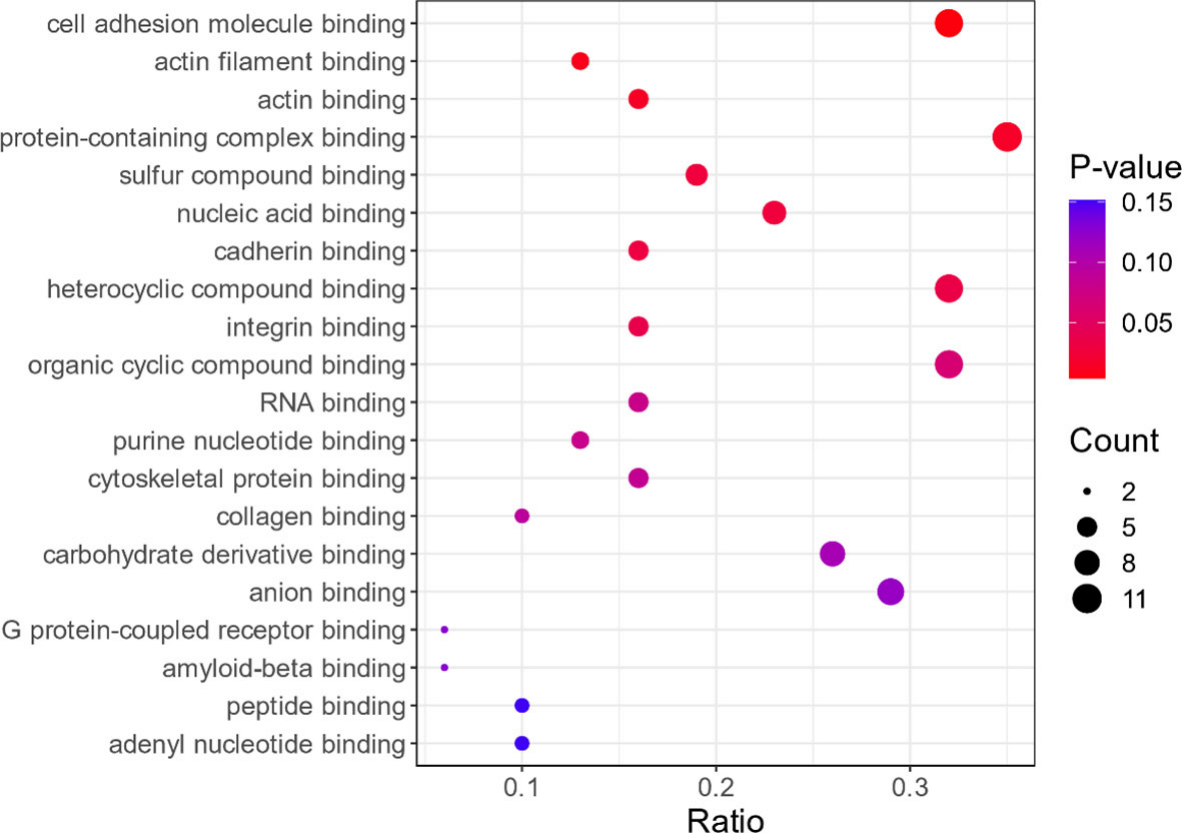
Bubble plot Gene Ontology (GO) enrichment analysis in terms of molecular function (only the top 20 are shown). Description is the same as in **Figure 2**.

**Figure 4:**
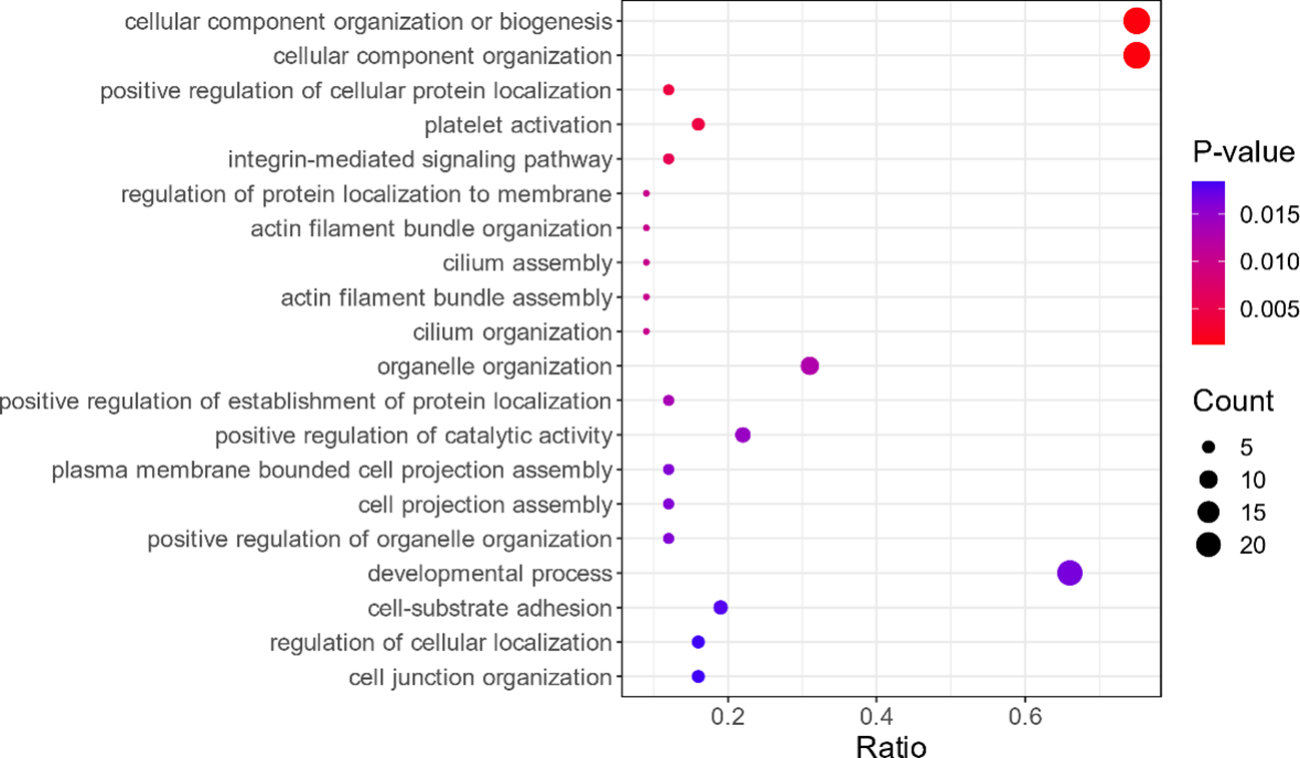
Bubble plot Gene Ontology (GO) enrichment analysis in terms of biological processes (only the top 20 are shown). Description is the same as in **Figure 2**.

In this study, GO enrichment analysis was performed for the 34 differential proteins identified. The results showed that, in terms of cellular components, the proteins are involved in non-membrane organelles and intracellular non-membrane organelles (see **Figure 2**). In terms of molecular function, these proteins are mainly involved in the combination of cell adhesion molecules, protein-containing complexes, and heterocyclic compounds, as shown in **Figure 3**. In terms of biological processes, the proteins are involved in cellular component organization or biogenesis (see **Figure 4**).

#### 3.2.4 KEGG enrichment analysis of the differential proteins

**Figure 5:**
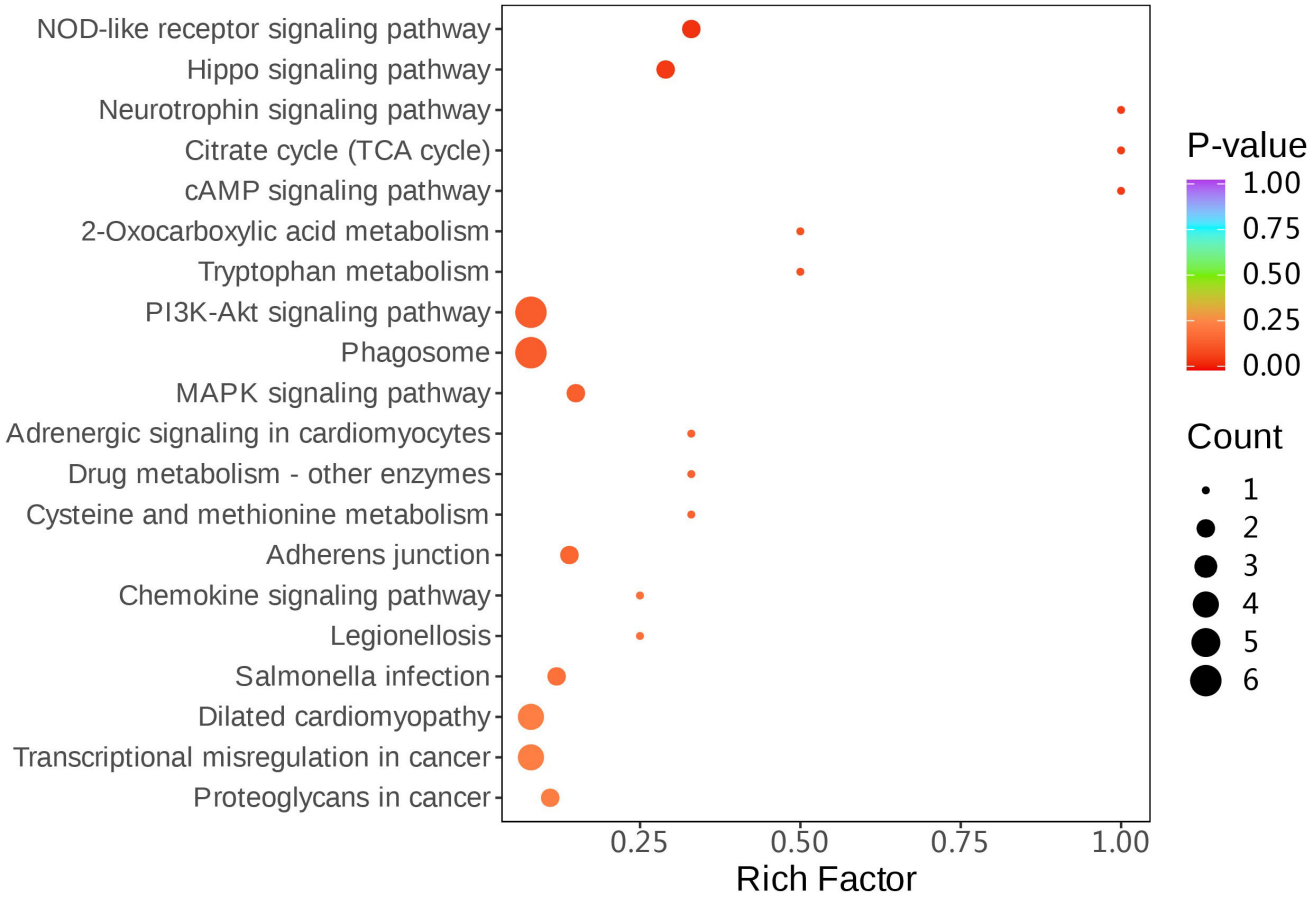
Kyoto Encyclopedia of Genes and Genomes (KEGG) enrichment bubble plot. The *abscissa* represents the ratio of the number of differential proteins in the corresponding pathway to the number of all proteins identified in that pathway. The higher the value, the higher the enrichment of the differential proteins in the pathway. The *color of the dot*, ranging from blue to *red*, indicates the corrected *p*-value of the hypergeometric test. The *redder the color*, the smaller the value, indicating greater reliability of the test and more statistically significant results. The *size of the dot* indicates the number of differentially expressed proteins in the corresponding pathway. The *larger the dot*, the more differentially expressed proteins within the pathway.

In addition, KEGG enrichment analysis was performed for the 34 differential proteins identified. The results showed that the significantly expressed differential proteins in the schizophrenia group were mainly enriched in the PI3K–Akt signaling pathway and the mitogen-activated protein kinase (MAPK) signaling pathway compared with the control group. In addition, the key proteins of the PI3K–Akt signaling pathway were A0A075B6R2, A0A0C4DH33, P17301, P31946, P49747, and P63104, while the key proteins in the MAPK signaling pathway were P21333 and P61224, as shown in **Figure 5**, **Supplementary Table S1**.

#### 3.2.5 Protein interaction analysis

**Figure 6:**
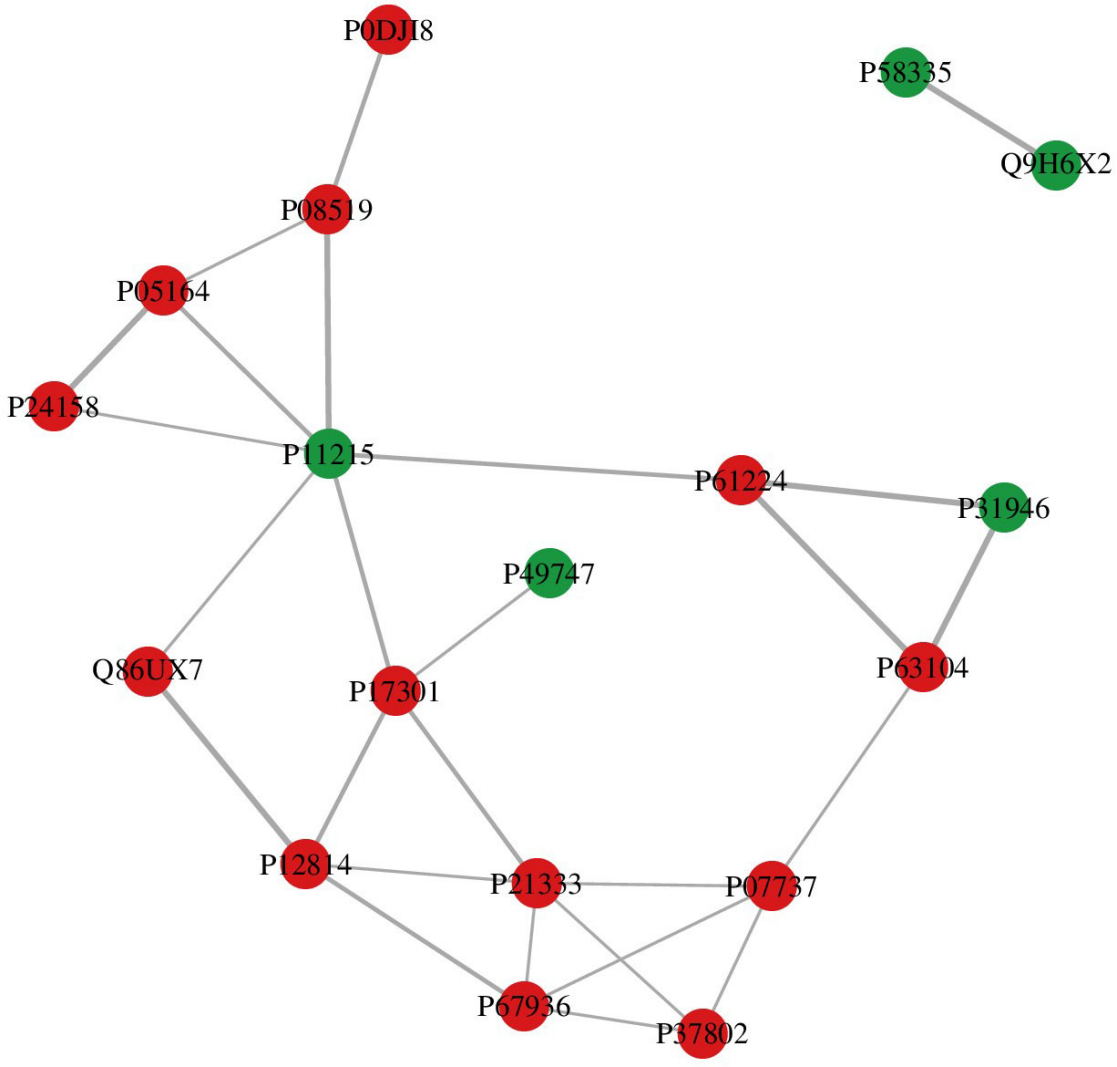
Protein interaction network diagram.

In this study, the STRING protein interaction database was used to analyze the protein–protein interactions (PPIs). If the corresponding species is available in the database, the sequences of the corresponding species were directly extracted; otherwise, the sequences of the closely related species were extracted. Subsequently, the differential protein sequences were compared with the extracted sequences using BLAST to obtain the corresponding interaction information, and a network graph was constructed using R (igraph). According to the network diagram, the main crossing node proteins were integrin alpha-M (P11215), profilin-1 (P07737), filamin A (P21333), P58335, Q9H6X2, P49747, P31946, P0DJI8, P08519, P05164, P24158, Q86UX7, P12814, P17301, P67936, P37802, P63104, and P61224 (see **Figure 6**, **Supplementary Table S1**).

### 3.3 Correlation of the differential proteins with mental symptoms, gender, and antipsychotic drug dosage

In the patient group, Spearman’s correlation analysis showed no significant correlations between the five candidate proteins and the Positive and Negative Syndrome Scale (PANSS) total score, positive score, negative score, and general pathological score (*p* > 0.05). There were also no significant correlations of the five candidate proteins with gender and antipsychotic drug dosage. The dosages of the antipsychotic drugs were converted into chlorpromazine equivalents (**Table 4**).

**Table 4:** Correlation analysis between the candidate proteins and the mental symptoms in the patient group.

	L1	Integrin alpha-M	Alpha-actinin-1	Filamin A	Profilin-1
PANSS total score	*r*=−0.046 *p*=0.761	*r*=−0.087 *p*=0.567	*r*=−0.069 *p*=0.649	*r*=−0.080 *p*=0.598	*r*=−0.027 *p*=0.858
PANSS positive score	*r*=−0.122 *p*=0.418	*r*=−0.083 *p*=0.582	*r*=−0.105 *p*=0.489	*r*=−0.193 *p*=0.198	*r*=−0.125 *p*=0.409
PANSS negative score	*r*=0.070 *p*=0.642	*r*=−0.123 *p*=0.415	*r*=−0.017 *p*=0.913	*r*=0.012 *p*=0.938	*r*=−0.071 *p*=0.639
PANSS general pathological score	*r*=−0.106 *p*=0.485	*r*=−0.061 *p*=0.687	*r*=−0.015 *p*=0.922	*r*=−0.099 *p*=0.514	*r*=0.064 *p*=0.675
Sex	*r*=0.025	*r*=−0.134	*r*=0.168	*r*=0.029	*r*=0.037
	*p*=0.819	*p*=0.212	*p*=0.116	*p*=0.786	*p*=0.728
Chlorpromazine (mg)	*r*=0.135	*r*=0.048	*r*=−0.008	*r*=0.041	*r*=0.195
	*p*=0.372	*p*=0.752	*p*=0.958	*p*=0.786	*p*=0.193

*PANSS*, Positive and Negative Syndrome Scale.

## 4 Discussion

Schizophrenia is a chronic and persistent disease whose pathological mechanism is still unclear. The majority of patients with this condition experience social withdrawal and a gradual decline, eventually leading to adverse outcomes of mental disability. Due to the lack of clear diagnosis and early and dynamic monitoring of the patient’s status evaluation system, it is impossible to truly achieve the whole course of treatment and management. This case–control study aimed to determine molecular biomarkers related to the pathology, diagnosis, or treatment of schizophrenia through mass spectrometry-based proteomic analysis.

### 4.1 Differential protein level profiles in the plasma of patients with schizophrenia

In this study, a total of 7,633 peptides and 668 proteins were identified in patients with schizophrenia and in healthy volunteers. Through quantitative analysis of the differential proteins, a total of 34 differential proteins were screened. It should be noted that the differential testing used nominal *p*-values without multiple testing correction; therefore, the number of differentially expressed proteins may be reduced after controlling for the FDR. The protein interaction analysis showed that the cross-node proteins were integrin alpha-M, profilin-1, and filamin A, which are mainly related to cytoskeleton, synapse, and immunity. This highlights a possible key role for these three proteins in schizophrenia regulation, which warrants further investigation.

Functional enrichment analysis of the differential proteins was performed to determine the GO entries involved in the differential proteins. It was found that, in terms of cellular components, the differential proteins are mainly related to the non-membrane organelles and intracellular non-membrane organelles. In terms of molecular functions, the differential proteins are mainly involved in binding cell adhesion molecules, protein-containing complexes, and heterocyclic compounds. In terms of biological processes, the differential proteins are mainly involved in cellular component organization or biogenesis. Studies have shown that cellular adhesion molecules on endothelial cells may promote leukocyte binding and the transendothelial migration of inflammatory factors, suggesting that endothelial inflammation may be involved in the pathogenesis of schizophrenia (27). It has also been suggested that, in schizophrenia, metabolic syndrome may induce neuroinflammatory changes in the brain through the endothelial components of peripheral inflammatory processes, such as intercellular adhesion molecules and vascular or neural cell adhesion molecules, although further confirmation is needed (28). Our research revealed that the differential proteins are involved in the binding of cell adhesion molecules and other processes, such as cell component organization and biological development. This further confirms the role of cell adhesion molecules and other elements in the pathogenesis of schizophrenia.

At the same time, KEGG functional enrichment analysis was performed for the identified differential proteins, which revealed that the proteins with significant differences in their levels between the schizophrenia group and the control group are mainly enriched in the mitogen-activated protein kinase (MAPK) signaling pathway, which had been confirmed by Western blot studies on brain tissue (29). It is also consistent with the results of the pathways enriched by transcriptomics (30). For example, the ERK/MAPK signaling pathway has been reported to have abnormal levels of several components in schizophrenia neurons (31) and was also disrupted in human-induced pluripotent stem cell (hi-PSC) neurons derived from schizophrenia patients with 22q11.2 deletion (27). Our results further elucidated the important role of the MAPK signaling pathway in the neuropathology of schizophrenia in the Chinese population, which warrant further investigation. Moreover, in the schizophrenia group, the significantly expressed differential proteins were also enriched in the PI3K–Akt signaling pathway. A study has shown that the antipsychotic effects of aripiprazole and sertindole are partly attributed to the reduction of oxidative stress and the activation of the NRG1/ErbB4 and PI3K/Akt/mTOR signaling pathways (32). SEP-363856 (SEP-856), which is a new antipsychotic, may exert its antipsychotic effect in mice with schizophrenia-like behaviors induced by the MK-SI “dual-hit” model by promoting the restoration of synaptic plasticity, reducing the death of hippocampal neurons, decreasing the production of pro-inflammatory substances in the hippocampal region, and thereby initiating the PI3K/Akt/GSK-3β signaling cascade reaction (33). Olanzapine was found to promote adipogenesis by inducing glycolysis and activating the downstream PI3K–Akt signaling pathway (34). The above studies suggest that the PI3K–Akt signaling pathway may be closely related to the mechanism of antipsychotic drugs. As the majority of the patients with schizophrenia we enrolled were on medication, the enrichment of this pathway might be due to the effect of antipsychotic drugs. However, there are currently only a few studies on the relationship between this pathway and schizophrenia, and the specific association and mechanism still require further investigation.

### 4.2 Analysis of the plasma candidate protein markers associated with schizophrenia

We nominated five proteins (i.e., neural cell adhesion molecule L1, integrin alpha-M, alpha-actinin-1, filamin A, and profilin-1) based on their effect sizes, network centrality in the PPI analysis (**Figure 6**), and prior biological plausibility. It is speculated that they may be involved in the pathological process of the occurrence and development of schizophrenia. The subsequent discussion will focus on these five candidate proteins.

#### 4.2.1 Neural cell adhesion molecule L1

Neural cell adhesion molecule L1, whose coding gene is *L1CAM*, is a member of the immunoglobulin superfamily expressed in the nervous system, which plays a key role in axon growth, synapse formation, and neuronal migration (35). The L1 family of cell adhesion proteins include L1, CHL1 (a close homolog of L1), NrCAM, and neurofascin. These molecules regulate neuronal development and neural networks, whose defects have been linked to various neurological diseases (36). L1 and CHL1 are more studied and the most likely associated with schizophrenia. A Japanese study found that *L1CAM* gene mutation of some base pairs are positively correlated with male schizophrenia, but has nothing to do with female schizophrenia (37). Our research did not reveal any such gender differences. This also indicates the direction that future research should focus on. In other studies, the *L1CAM* immunoreactive protein was reduced in the cerebrospinal fluid of patients with schizophrenia compared with healthy controls (38, 39), while the study of the brain tissue after death failed to show significant changes in the *L1CAM* protein in schizophrenia (40, 41). Our study found that the level of L1 was upregulated in patients with schizophrenia compared with healthy controls, which is inconsistent with existing results. This may be due to the fact that the samples in our study were derived from peripheral blood, which is different from the cerebrospinal fluid and brain tissue. In addition, some studies have suggested that *CHL1* is a candidate gene for schizophrenia (42). However, our study did not find differential levels of the L1 homologues, which may be due to the small sample size and because it is not a genomic study. At present, some scholars believe that schizophrenia is a synaptic disorder characterized by the functional disruption of synaptic regulatory proteins (43). The L1 family is associated with synapse formation and plasticity by mediating spinal pruning during development and playing an important role in the regeneration of the nervous system (43). It is also involved in the formation of the myelin sheath that surrounds many axons (44). In conclusion, the present studies suggest that L1 is involved in the pathogenesis of schizophrenia. However, the association between L1 in peripheral blood and schizophrenia needs to be verified by more studies.

#### 4.2.2 Integrin alpha-M

Integrin alpha-M, also known as CD11b, is a component of complement receptor 3 (CR3) and an activation marker of the microglia (45). Its encoding gene is *ITGAM*. Our study found that CD11b was downregulated in patients with schizophrenia, which is consistent with findings in animal models of schizophrenia (46). However, other studies have shown the microglia in experimental rats to exhibit high levels of CD11b immunoreactivity (45). The long-term activation of the microglia causes changes such as neuronal degeneration, reduced neurogenesis, white matter abnormalities, cell apoptosis, and brain damage, which is also one of the possible pathophysiological mechanisms of schizophrenia (47). There is no consensus on the level of CD11b in the disease, which may be due to the use of different animal models of schizophrenia. However, in the same Poly real I:C maternal immune activation model, Manitz et al. (48) found that the CD11b level was significantly lower than the control group in the adult offspring and was more obvious in male offspring, while Hui et al. (49) found a higher level in female offspring. However, our research did not reveal any gender differences. These differences may be related to factors such as race and sample size, suggesting that there might be gender differences among these proteins. Further research is necessary in the future. This model, which simulates the association between prenatal infection and schizophrenia in later life, has become one of the most powerful developmental models of schizophrenia. The binding of complement C3 on neurons to CR3 on the microglia results in the engulfing of developing synapses, and the microglia contribute to the plasticity and stability of the central nervous system (48, 50). The reduced CD11b/CR3 level may be associated with an impaired synaptic surveillance and a reduced ability to eliminate abnormal synapses (48). CR3 shares an expression pattern with C3 in the developing brain, and CD11b is also significantly correlated with the expression levels of C3 (51). Interestingly, our findings showed an abnormal downregulation of CD11b in schizophrenia, which is consistent with other research. Therefore, this indicates that CD11b deserves further exploration as a candidate protein marker for schizophrenia, as well as the relationship between the microglia, complement, and synapses.

#### 4.2.3 Alpha-actinin-1

Alpha-actin is one of the first muscle cell molecules with the function of crosslinking with actin filament bodies described more than 50 years ago (52) and later found in non-muscle cells (53), which is a ubiquitous cytoskeletal protein in eukaryotes. There are four known types of alpha-actin—1, 2, 3, and 4—with 1 and 4 being expressed in non-muscle cells, which may be important molecules in immune response (54). Alpha-actin is a scaffold that integrates signaling molecules at adhesion sites and can promote the aggregation of adhesion molecules at specific sites (55), indicating that they play an important role in the connection of the cytoskeletal structures to the plasma membrane (56). Yan et al. (57) found that the synaptic actin cytoskeleton is impaired in schizophrenia, possibly leading to a reduced spinal stability and, ultimately, spinal loss. The pathogenic processes and the molecular mechanisms of cell type-specific alterations in actin dynamics may produce cortical dendritic spine defects as upstream signaling pathways, resulting in subsequent striatal dopamine hyperfunction and the emergence of schizophrenia (57). Studies of autopsy brain tissue found a reduced actin polymerization in the anterior cingulate cortex in elderly patients with schizophrenia, and it has been confirmed that the density of dendritic spines and the synaptic plasticity are reduced in schizophrenia (58). However, our study found that the upregulation of alpha-actin-1 in schizophrenia, probably due to the different sources of organization, the number of actin isoforms, and the autopsy study, did not specify the type of actin. Schizophrenia is associated with a reduced dendritic spine density and altered spine morphology in several regions of the human brain, particularly layer 3 of the neocortex (59). As actin is highly enriched in dendritic spines and is the major cytoskeletal protein in dendritic spines that controls spine morphogenesis and spine plasticity (60), actin isoforms may reflect the pathological process of schizophrenia to a certain extent. More studies using peripheral blood are needed to confirm this in the future.

#### 4.2.4 Filamin A

Filamin A is an actin-binding protein with a molecular weight of 280 kDa (61) and is also a widely expressed cytoskeleton-related protein. It plays an important role in the regulation of cell morphology and movement (62). Our study found an upregulation of filamin A compared with the controls. There are relatively few studies on the levels of filamin A in schizophrenia. Filamin A has been reported to interact with the third cytoplasmic loop of dopamine 2 (D2) and dopamine 3 (D3) receptors, suggesting a molecular mechanism by which cytoskeletal protein interactions regulate D2 and D3 receptor signaling (62, 63). Filamin A and spinophilin link D2 receptors to the actin cytoskeleton and can serve as scaffolds to assemble the various components of the D2 receptor signaling complex, capable of promoting D2 receptor aggregation (64). At the same time, filamin A is also thought to be required for neuronal migration in humans. Moreover, filamin A interacting protein and filamin A may be involved in cortical development, representing one of the interlinked protein networks in psychiatric disorders (65). In addition, the dopamine hypothesis is well known as one of the widely accepted pathogeneses of schizophrenia, and the dopamine signaling pathway is regulated by filamin A. Our research findings indicate that filamin A may play a certain regulatory role in schizophrenia. However, the specific mechanism remains unclear and requires further investigation.

#### 4.2.5 Profilin-1

Profilin was one of the first actin-binding proteins identified in the 1970s (66), catalyzing actin activity in a concentration-dependent manner: actin polymerization is prevented at high concentrations, while it appears to be promoted at low concentrations (67). There are four isoforms of profilin identified so far, of which profilin-1 is the most highly regarded due to its role in the cytoskeleton and in cell signaling and its link to cancer and vascular hypertrophy. It is present in almost all tissues and cells, including platelets, lymphocytes, and glia, among other (66). Profilin-1 also plays a role in shaping the synaptic structure and is an important regulator of synaptic plasticity. Its deficiency may adversely affect neuronal development (66, 68). Researchers found that profilin-1 was upregulated in the mouse hippocampus via proteomic analysis in a mouse model of prenatal stress, suggesting that it could interfere with cytoskeleton remodeling during development (68). Our study also found profilin-1 to be upregulated compared with the controls, which is consistent with the results in mice. However, there are relatively few studies on the association of the level of profilin-1 in peripheral blood with schizophrenia. Hence, more human studies are needed to verify their association in the future.

In our research, the differential proteins neural cell adhesion molecule L1, integrin alpha-M, alpha-actinin-1, filamin A, and profilin-1 are nominated as schizophrenia-associated candidates. Orthogonal verification [targeted parallel reaction monitoring (PRM)/ELISA] and replication in an independent cohort will establish their robustness and generalizability. Interestingly, all these candidate proteins are related to synaptic functions. Proteomic and genomic evidence implicates the postsynaptic density in schizophrenia (69). One study (70) on deep and unbiased proteomic analysis of the lateral prefrontal cortex synapses revealed that, in the synapses of patients with schizophrenia or bipolar disorder, the proteins related to autophagy and certain vesicle transport pathways were upregulated, while the proteins related to synaptic, mitochondrial, and ribosomal functions were downregulated. Furthermore, in the synaptic proteome of mutant mice with defective *Akap11*, a newly discovered common risk gene for schizophrenia and bipolar disorder, a similar dysregulation was observed in some of the same pathways (70). Another study using postmortem samples from patients with schizophrenia revealed that, compared with normal controls, the synaptic protein profile underwent a powerful and highly coordinated reorganization, and the synaptic levels of postsynaptic proteins changed significantly (71). All of the above studies demonstrated a close correlation between the synaptosome or the postsynaptic density proteomes and schizophrenia, further supporting our research findings. Although our research sample was taken from peripheral blood, the consistent results with those from central tissues precisely indicated that changes in certain synaptic-related proteins in the peripheral blood might reflect the changes in central proteins. This further verified the potential of these proteins as biomarkers for schizophrenia. However, the differences in the protein levels between the central and peripheral regions still require further investigation. Our research did not find any association between the five proteins and the severity of schizophrenia, and it is possible that these proteins represent characteristic changes of schizophrenia and are not related to the progression of the disease. This may also be related to the fact that the patients had already received treatment and that their conditions had become chronic. Future studies could further investigate the relationship between these five proteins and schizophrenia, particularly in the case of first-episode schizophrenia, and conduct follow-ups, which might yield more meaningful results.

There are several limitations in this study. Firstly, this study has a cross-sectional design and could not capture the protein levels before and after drug treatment. Secondly, incomplete control for potential confounding factors, such as medication use, coexisting conditions, or other clinical features, may have affected the blood protein levels, and there may be uncertainty as to whether the identified blood proteins reflect characteristic or state markers of schizophrenia. Thirdly, the five candidate proteins were selected *post hoc* from a broader differential list and were not validated by orthogonal assays or in an independent cohort. Targeted PRM- and ELISA-based verification followed by replication are required to substantiate these candidates. In addition, given that the GO and KEGG enrichment analyses did not perform FDR correction, these results should be interpreted with caution and validated in larger cohorts. Finally, although peripheral blood can reflect the physiological and pathological changes of any part of body to some extent, the biological interpretability of blood proteins in the context of central nervous system pathology still needs to be further elucidated.

## 5 Conclusions

This study suggests that proteins associated with the cytoskeleton, synapse, and immunity are related to schizophrenia, in particular neural cell adhesion molecule L1, integrin alpha-M, alpha-actinin-1, filamin A, and profilin-1, which are expected to be candidate protein markers for schizophrenia. The MAPK signaling pathway may be related to the pathology of schizophrenia, while the PI3K–Akt signaling pathway may be related to the efficacy and side effects of antipsychotic drugs.

## Data Availability

The datasets presented in this study can be found in online repositories. The names of the repository/repositories and accession number(s) can be found in the article/**Supplementary Material**.
