# Supplementary material for: Five differentially expressed proteins identified to serve as potential blood biomarkers for schizophrenia screening based on proteomics
Source: Front Psychiatry. 2026 Jan 7;16:1697383. doi: 10.3389/fpsyt.2025.1697383 (PMC12819645; doi:10.3389/fpsyt.2025.1697383)
Supplement: Supplementary file 1 [file DataSheet1.pdf]

Protein profile of PI3K-Akt signaling pathway:
The precursor ion spectrum of the peptide segments that may be produced by the proteins

A0A0C4DH33:

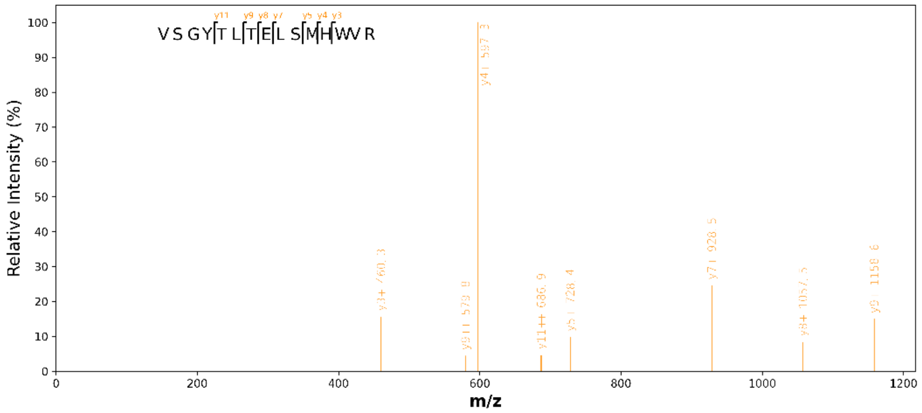

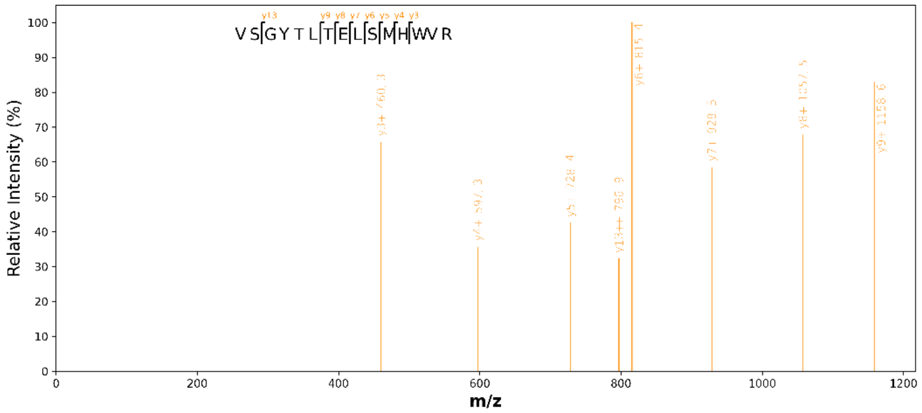

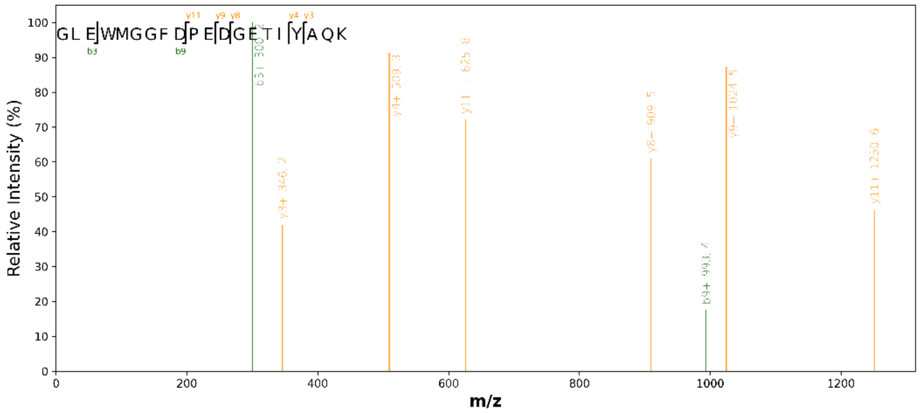

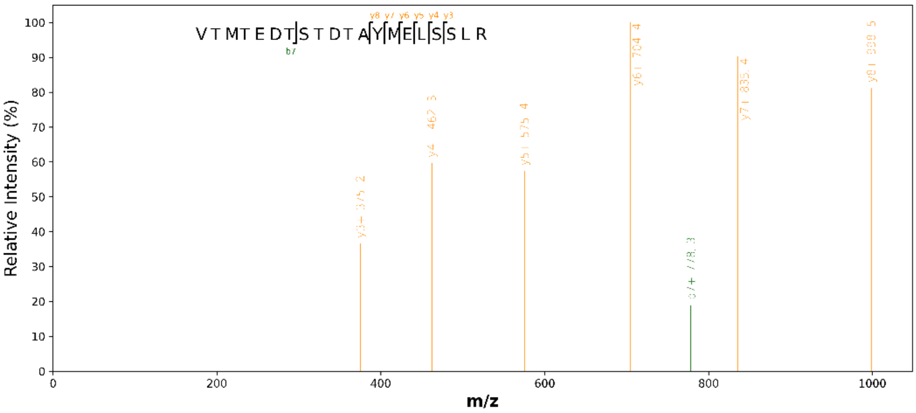

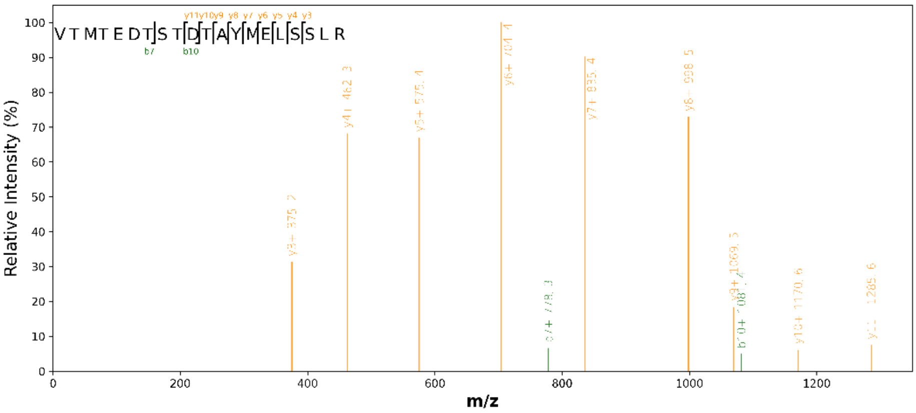

P31946:

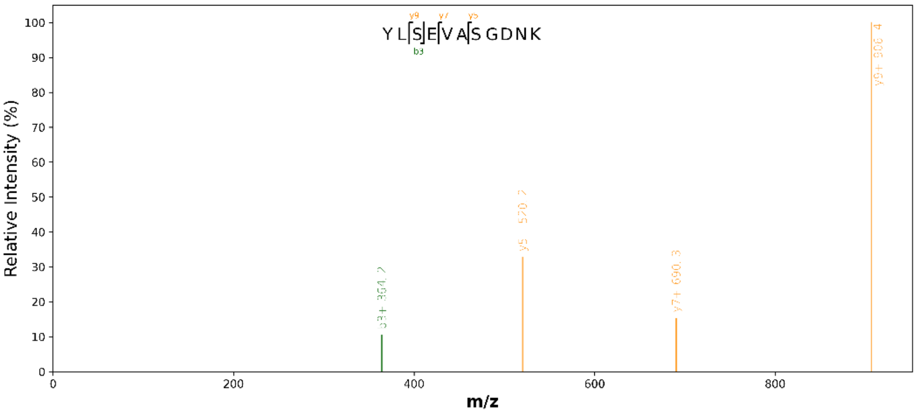

P49747:

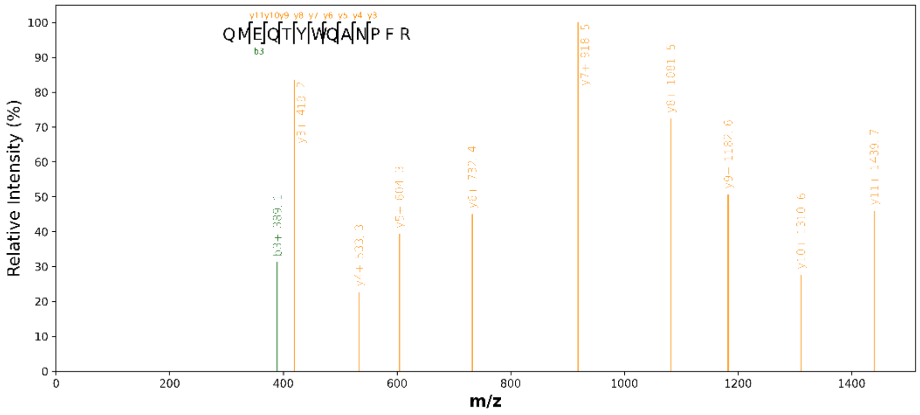
QMEQTYWQANPFR
y11 y10 y9 y8 y7 y6 y5 y4 y3
b3
Relative Intensity (%)
m/z
b3+ 389.1
y7+ 918.5
y9+ 1187.6

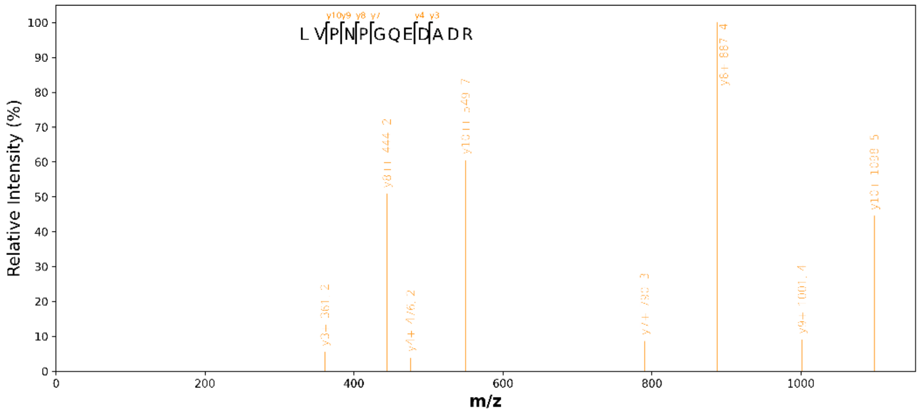
LVPNPGQEDADR
y10 y9 y8 y7 y4 y3
Relative Intensity (%)
m/z
y3+ 361.2
y8+ 444.2
y6+ 887.4
y9+ 1001.4

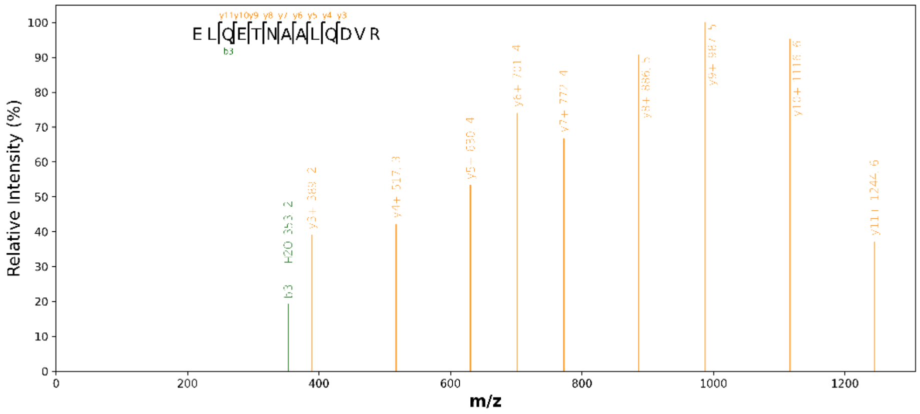
ELQETNAALQDVR
y11 y10 y9 y8 y7 y6 y5 y4 y3
b3
Relative Intensity (%)
m/z
b3
H2O 353.2
y3+ 389.2
y4+ 517.3
y5+ 630.4
y6+ 701.4
y7+ 772.4
y8+ 886.5
y11+ 1244.6

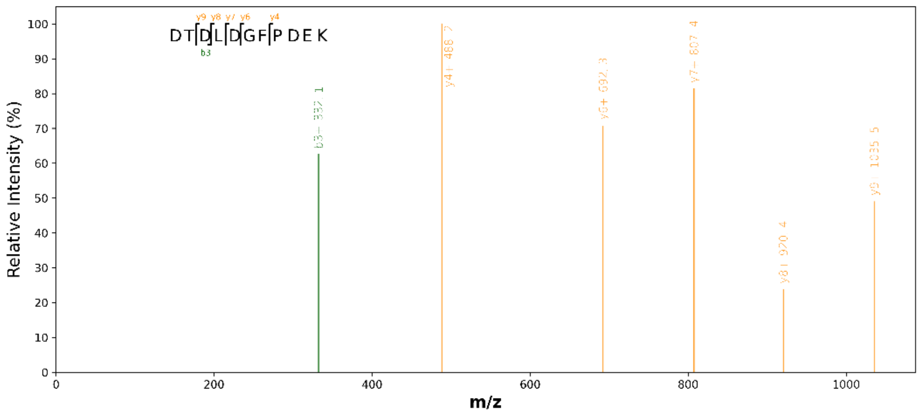
DTDLDGFPDEK
Relative Intensity (%)
m/z

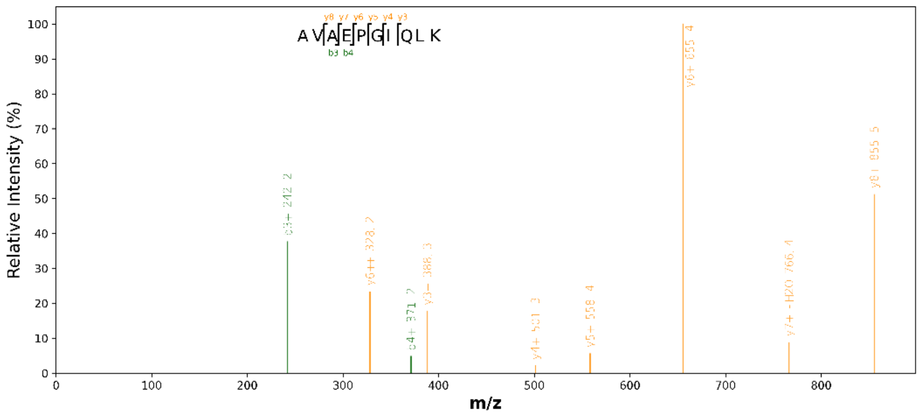
AVAEPGIQLK
Relative Intensity (%)
m/z

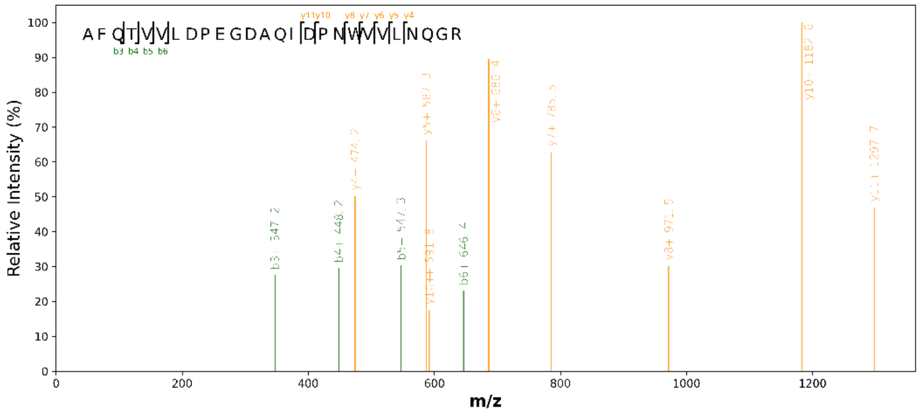
AFQTVVLDPEGDAQIDPNWVVLNQGR
Relative Intensity (%)
m/z

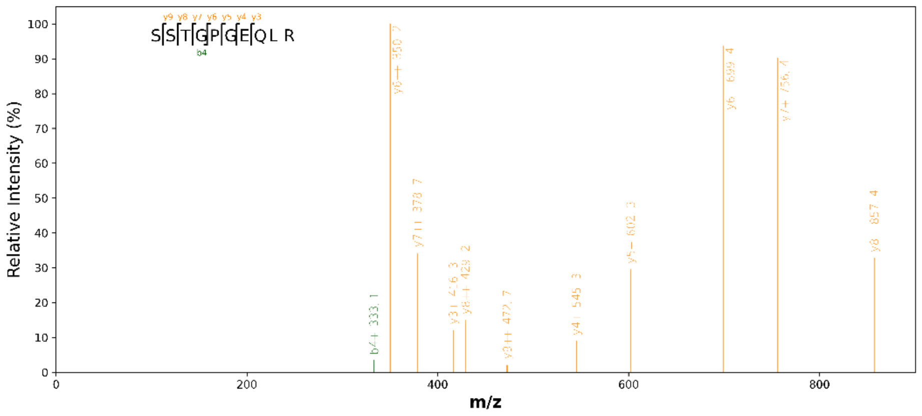
y9 y8 y7 y6 y5 y4 y3
S S T G P G E Q L R
b4
Relative Intensity (%)
m/z

P63105:

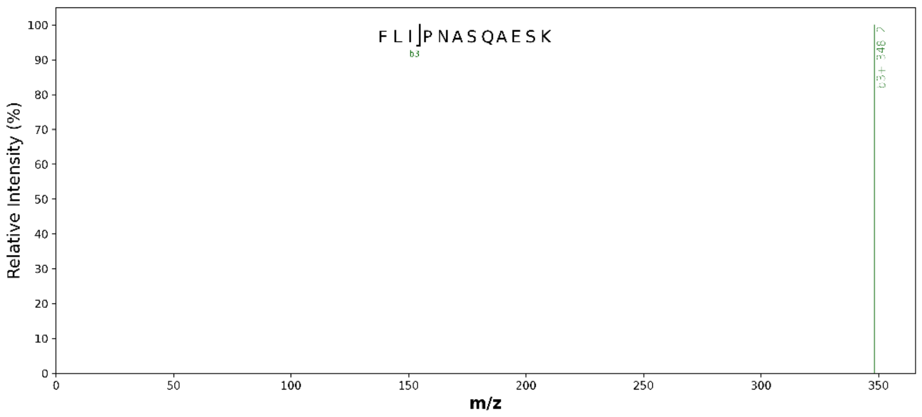
F L I P N A S Q A E S K
b3
Relative Intensity (%)
m/z

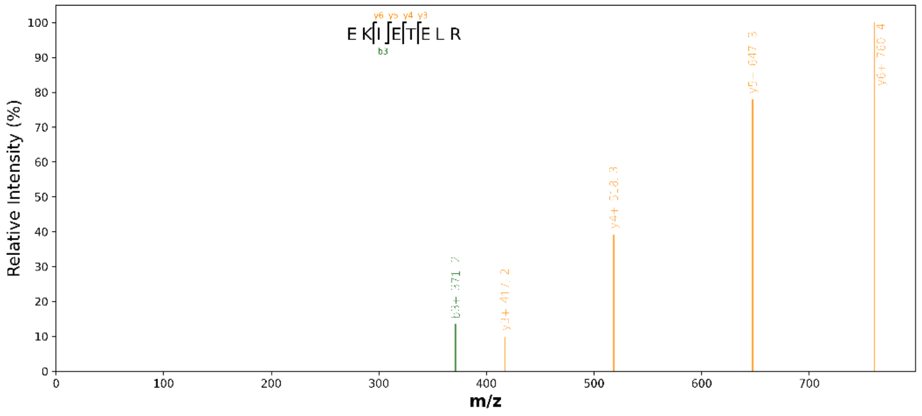
y6 y5 y4 y3
E K I E T E L R
b3
Relative Intensity (%)
m/z

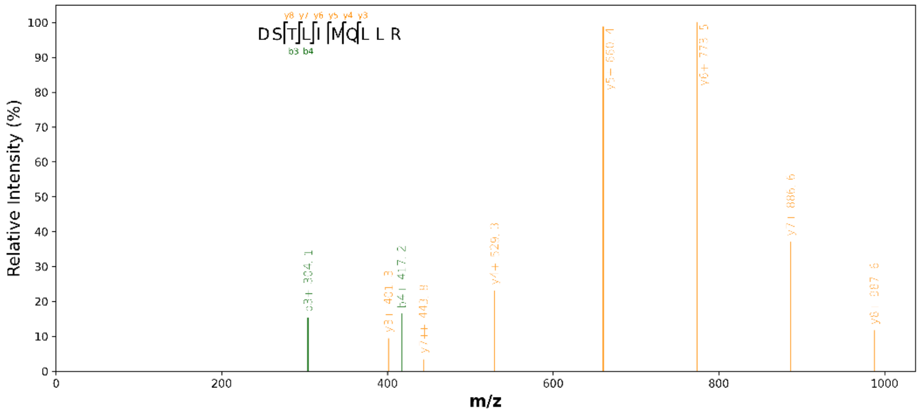
DS T L I MQ L L R
Relative Intensity (%)
m/z

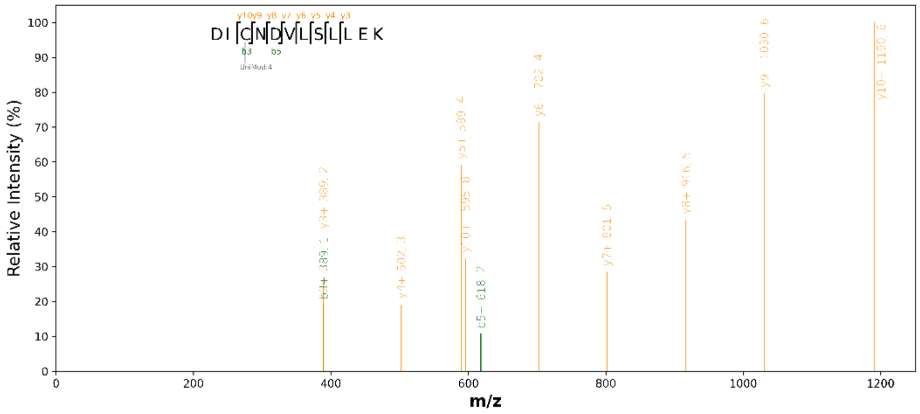
DI C N D V L S L L E K
Relative Intensity (%)
m/z

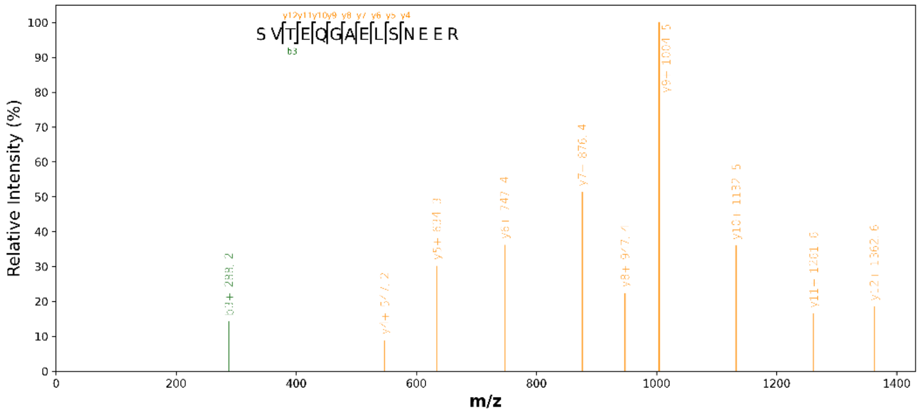
S V T E Q G A E L S N E E R
Relative Intensity (%)
m/z
