# Supplementary material for: Five differentially expressed proteins identified to serve as potential blood biomarkers for schizophrenia screening based on proteomics
Source: Front Psychiatry. 2026 Jan 7;16:1697383. doi: 10.3389/fpsyt.2025.1697383 (PMC12819645; doi:10.3389/fpsyt.2025.1697383)
Supplement: Supplementary file 2 [file DataSheet2.pdf]

Protein profile of MAPK signaling pathway:
The precursor ion spectrum of the peptide segments that may be produced by the proteins

P21333：

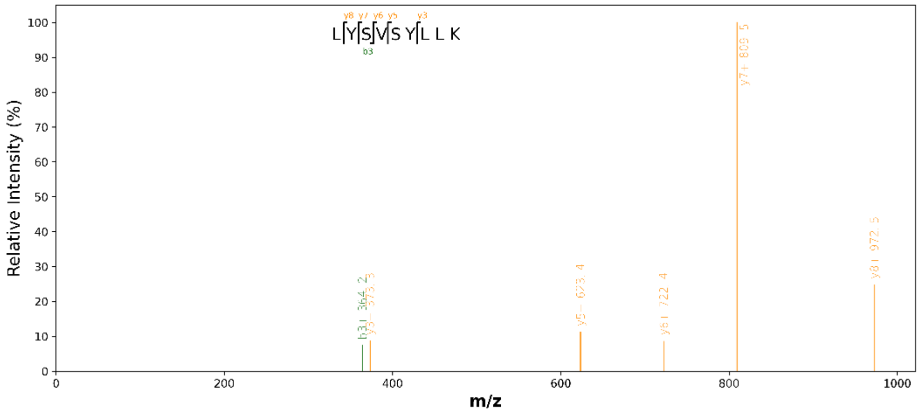

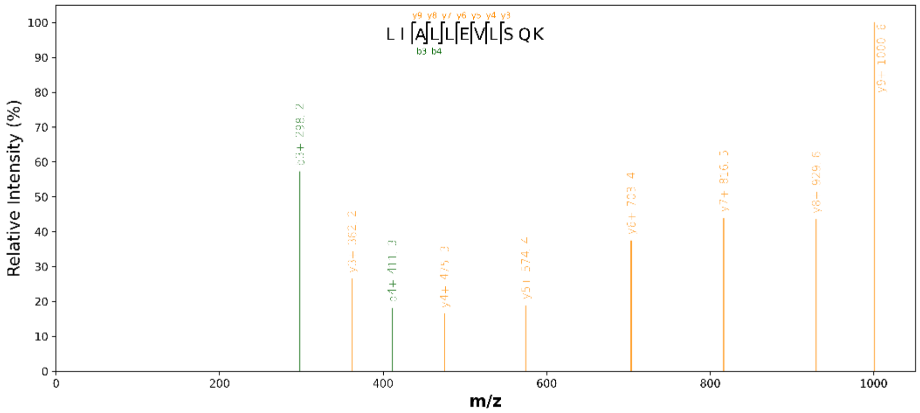

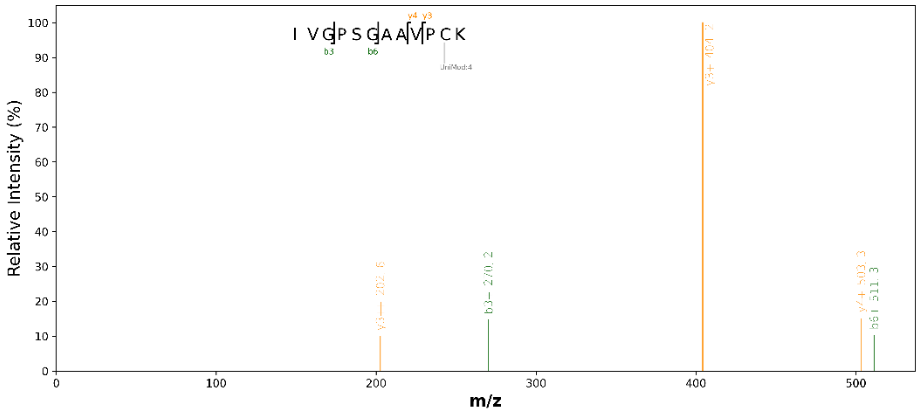

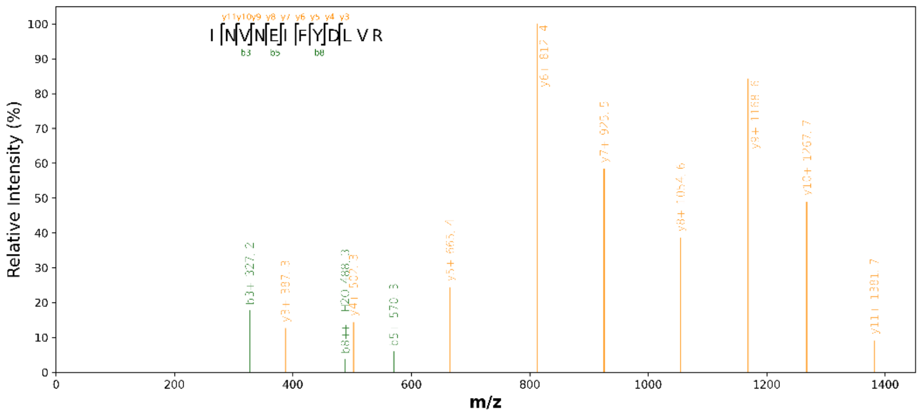
INVNEIFYDLVR
Relative Intensity (%)
m/z

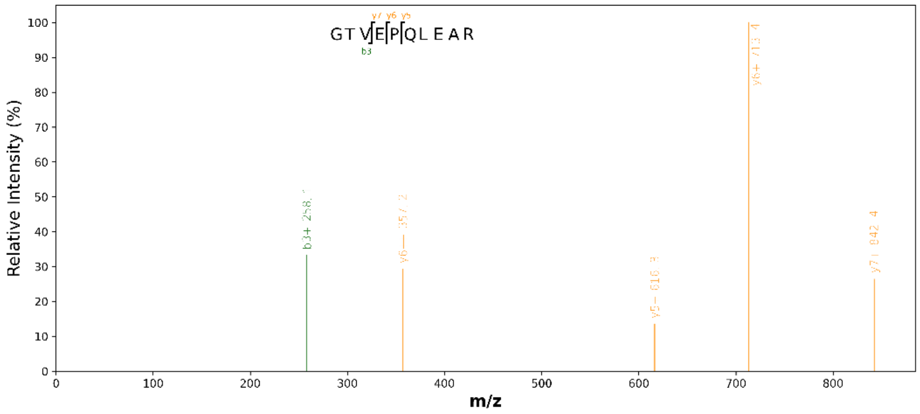
GTVEPQLEAR
Relative Intensity (%)
m/z

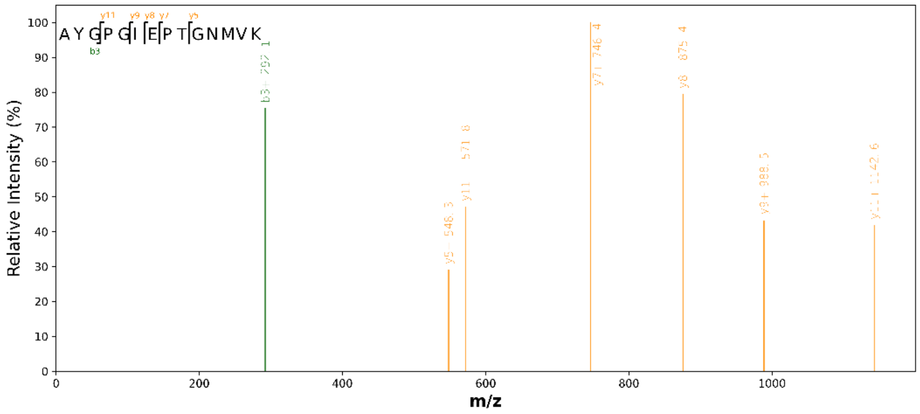
AYGPGIEPTGNMVK
Relative Intensity (%)
m/z

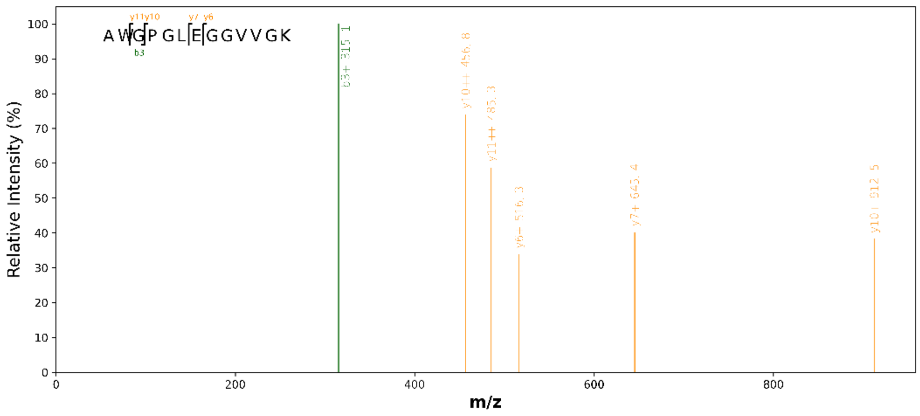
AWGPGLEGGVVGK
Relative Intensity (%)
m/z

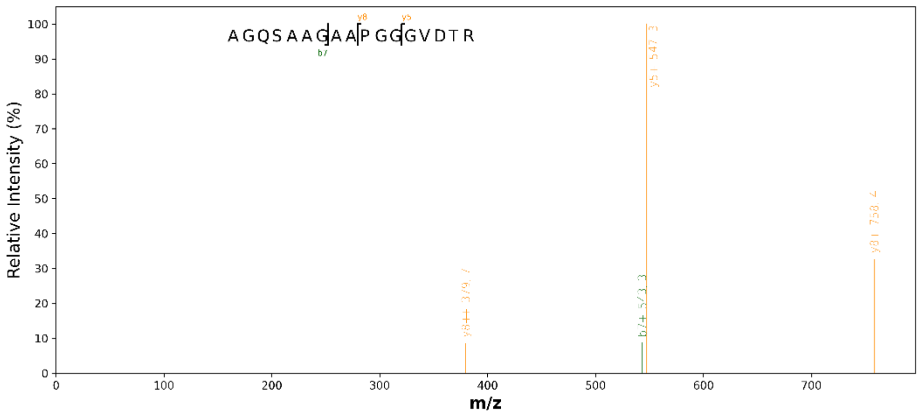
AGQSAAGAAPGGGVDTR
Relative Intensity (%)
m/z

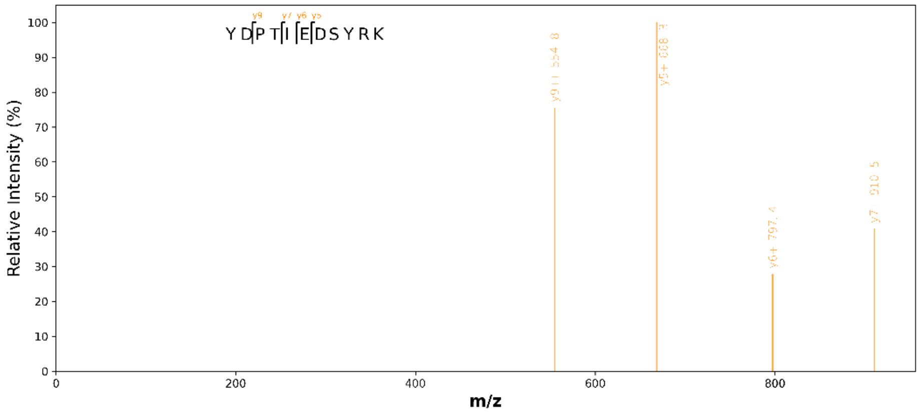
YDPTIEDSYRK
Relative Intensity (%)
m/z

P61224:

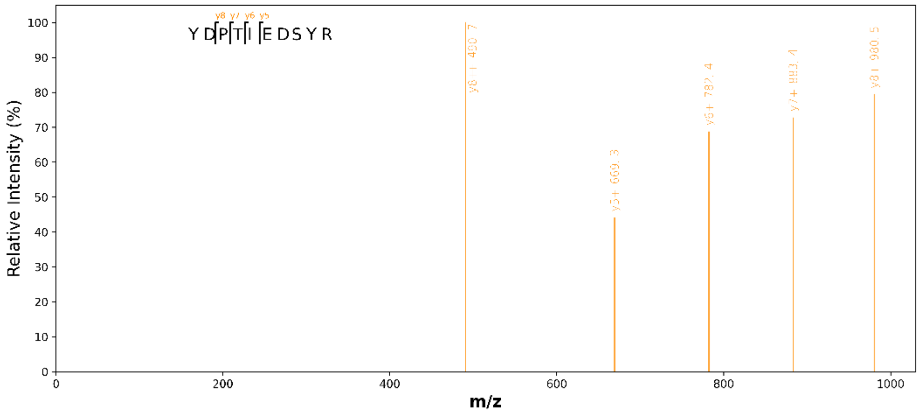
YDPTIEDSYR
Relative Intensity (%)
m/z

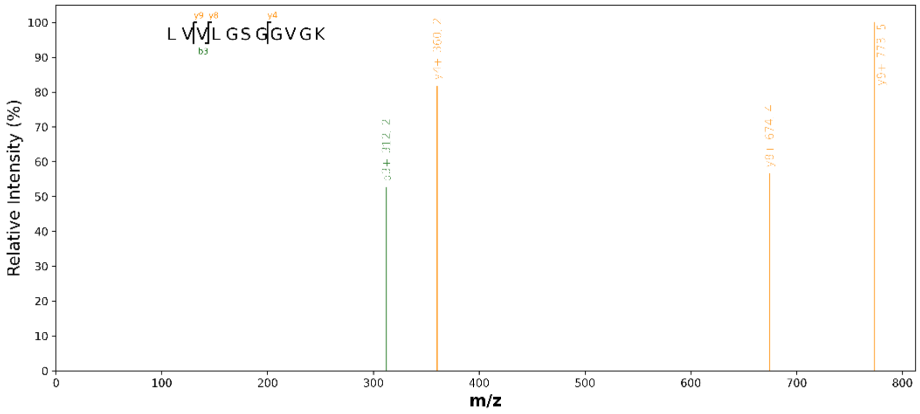
LVVLGSGGVGK
Relative Intensity (%)
m/z

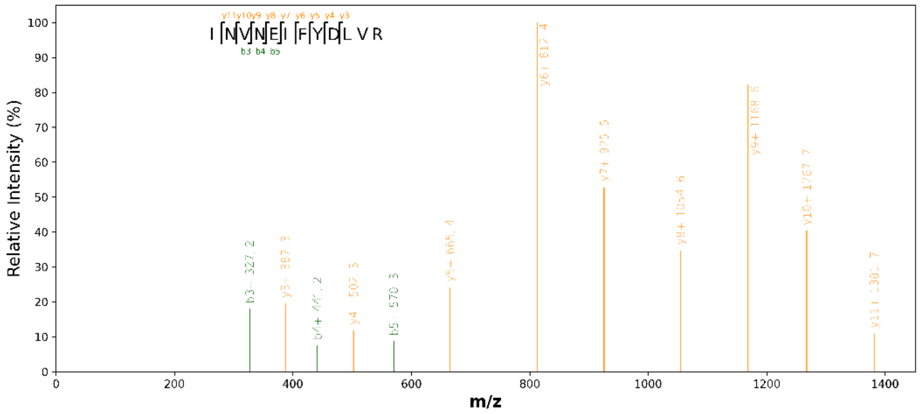
INVNEIFYDLVR
Relative Intensity (%)
m/z

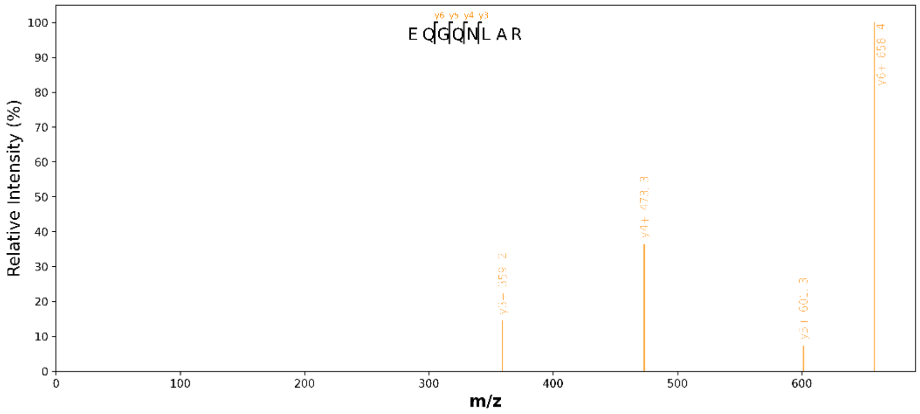

y6 y5 y4 y3
EQGQNLAR
Relative Intensity (%)
m/z
y4+ 473.3
y6+ 658.4

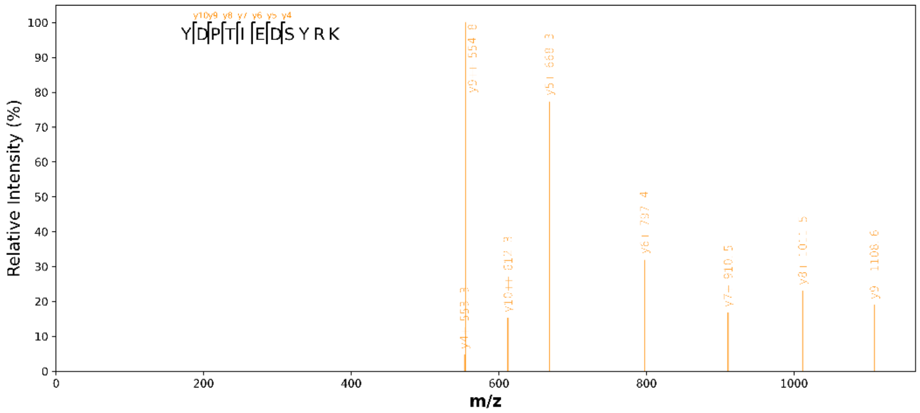

y10y9 y8 y7 y6 y5 y4
YDPTIEDSYRK
Relative Intensity (%)
m/z
y6+ 797.4
y7+ 910.5
y9+ 1108.6
